# The Anesthesia for Shoulder Surgery: Prospective.

**Published:** 2012-10-11

**Authors:** Antonio Izzo, Marco Baggi

**Affiliations:** Department of Anesthesiology, Beata Vergine Hospital, Mendrisio, Switzerland

**Keywords:** Shoulder Surgery, Brachial Plexus, Interscalene Ultrasound-guided Blockade

As largely explained by Lanna et al [1], the anaesthesia used for shoulder surgery offers many cues of reflection regarding the techniques that can be used.

At Beata Vergine Hospital, in Mendrisio, Switzerland, we carry out approximately 1000 shoulder surgeries per year. We are used to practice general anaesthesia with the auxiliary of a laryngeal mask added to ultrasound (US)-guided interscalene brachial plexus block (ISB).

The advantage of this combined method is a better miorelaxation of the muscular section where the operation takes part. A minor use of opiate and hypnotics, which are used only during induction with general anaesthesia, lead with low anaesthetic gas dosage. At least, the extension and the action of the local anaesthetic (LA) used for the ISB allows us to set up an adequate analgesic therapy before the pain gets unmanageable.

More recently the use of US-guided nerve blockade instead of a nerve stimulation-guided one has brought to an improvement regarding the ISB. The advantages are the visualization of the needle, the nervous structures and the LA injection during the procedure. The use of US-guided ISB reduces the number of attempts and allows the use of ultra-low volumes of LA. This procedure is more effective and successful in most cases and shows a reduction of the complications [2].

The completion of the anaesthetic technique of ISB does not influence surgical schedule as the US-guided nerve blockade concedes an important time saving.

Attention has to be focused on postoperative pain control. Even if the ISB ameliorates and lengthens the analgesia, after surgery some patients notice pain not controlled by medical drugs.

The application of a continuous infusion of local anaesthetics through an interscalene catheter seems the best available technique to achieve pain relief after shoulder surgery.

If the patient is discharged the second postoperative day, it requires a well-organized home health care in order to control the patient.

Even though our attention is focused on postoperative pain control, the medical drugs used at present, e.g. Paracetamol (1000 mg each 6 hours), Novalgin (1000 mg each 8 hours) and Morphin (3 mg on demand), are not able to control pain in all patients.

To such purpose we are planning an experimental study of US-guided interscalene brachial plexus anaesthesia, using a local anaesthetic (Prilocaine 1 %) with cortisone (Dexamethasone), in order to estimate if the extension of the analgesia given from the combination of these drugs, about 72 hours as we expect, allows us to achieve a better postoperative pain control.

Anaesthesiological procedures for shoulder surgery, more than other procedures, have gone through many changes given by the utilization of the US and by the current research of new methods in order to improve postoperative pain control.
